# Evaluation of junctional zone differential and ratio as possible markers of clinical efficacy in uterine artery embolisation of adenomyosis

**DOI:** 10.1186/s42155-024-00468-0

**Published:** 2024-07-18

**Authors:** Kolos Turtóczki, Hyunsoo Cho, Sorour Dastaran, Pál N. Kaposi, Zoltán Tömösváry, Szabolcs Várbíró, Nándor Ács, Ildikó Kalina, Viktor Bérczi

**Affiliations:** 1 Medical Imaging Centre, Semmelweis University, Üllői Út 78/a VIII. Korányi Sándor Utca 2., Budapest, 1082 Hungary; 2 Department of Obstetrics and Gynecology, Semmelweis University, VIII. Üllői Út 78/A, Budapest, 1082 Hungary

**Keywords:** Adenomyosis, Embolization, MRI, Clinical efficacy, Safety

## Abstract

**Background:**

Uterine artery embolisation is a recommended method of adenomyosis treatment with good clinical results. Changes in uterine volume and maximal junctional zone thickness (JZmax) after embolisation are thoroughly analyzed in the literature. In contrast changes in other suggested morphological diagnostic markers of adenomyosis (junctional zone differential / JZdiff—and junctional zone ratio / JZratio) are rarely evaluated. This single-centre retrospective study aimed to analyse the changes in morphological parameters used for the MR imaging diagnosis of adenomyosis (including JZdiff and JZratio) after UAE. Clinical effectiveness and safety were also analysed.

**Materials and methods:**

Patients who underwent UAE for pure adenomyosis from Jan 2008 to Dec 2021 were evaluated. Adenomyosis was diagnosed based on JZmax, JZdiff, and JZratio measured on MR imaging. To assess clinical efficacy, the numerical-analog-quality-of-life (QoL) score was routinely obtained from patients at our centre. MRI morphological data were analysed. Statistical analysis was conducted using Wilcoxon signed-rank test, uni- and multivariate regression models, Pearson product-moment correlation, and Kruskal–Wallis tests.

**Results:**

From our database of 801 patients who underwent UAE between Jan 2008 to Dec 2021, preprocedural MR images were available in 577 cases and, 15 patients had pure adenomyosis (15/577, 2.6%). Uterine volume, JZmax, and JZdiff decreased significantly after UAE; QoL score increased significantly. A significant correlation was found between QoL change vs. JZmax and JZdiff change. Permanent amenorrhoea and elective hysterectomy 5 years after UAE were both 7.1%.

**Conclusion:**

Change of JZdiff after UAE in adenomyosis is a potential marker of clinical success. UAE is a clinically safe and effective treatment for adenomyosis.

**Graphical Abstract:**

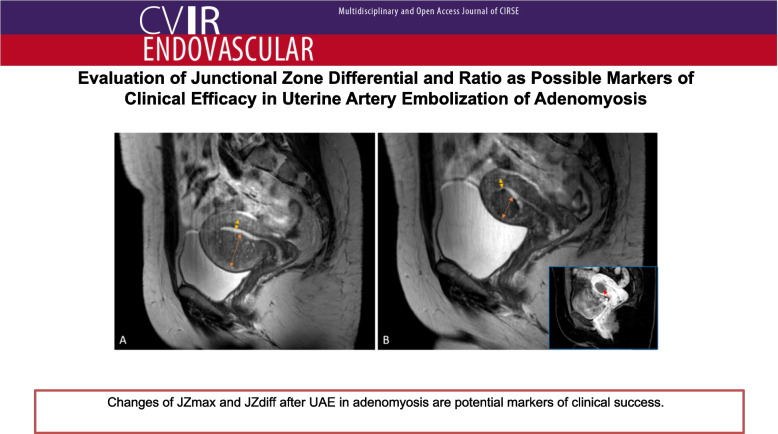

## Introduction

Adenomyosis is characterised by the presence of heterotopic endometrial glands and stroma deep within the myometrium with adjacent myometrial hyperplasia [1]. Heavy menstrual bleeding, pain, and fertility issues are the most common symptoms affecting 2/3 of premenopausal women with adenomyosis [2]. Magnetic Resonance (MR) imaging is currently used as a second-line imaging technique for adenomyosis after transvaginal ultrasound, as it has higher sensitivity and specificity, and lower operator dependence [3]. Diagnosis of adenomyosis on MRI is based on identifying direct and indirect signs. T1 or T2 hyperintense submucosal microcysts are considered the only direct sign of the disease representing ectopic endometrial glandular and stromal components displaced in the inner myometrium [4]. For a long time, the main indirect diagnostic criterion for the disease was maximal junctional zone thickness (JZmax) > 12 mm; however, due to the conflicting values for the sensitivity and specificity of this threshold, other indirect signs were introduced [5]. Junctional zone differential (JZdiff) is the difference between the maximal and the minimal thickness of the junctional zone. The junctional zone ratio (JZratio) is the ratio of the junctional zone thickness and the myometrial thickness at the location of maximal JZ thickness [6].

Uterine artery embolisation (UAE) has been proposed as a minimally invasive alternative to hysterectomy in the treatment of patients with symptomatic adenomyosis [7–10]. A decrease in uterine volume and JZmax after embolisation is a well-documented finding in the literature [9]. However, only scarce data is available regarding the change and possible predictive role of JZdiff and JZratio [11].

This single-centre retrospective study aimed to assess the change in MR morphological parameters after UAE in adenomyosis and to analyse the relation of morphological and clinical parameters. Long-term follow-up information is also provided.

## Materials and methods

All procedures performed in this study involving human participants were in accordance with the ethical standards of the institutional and/or national research committee and with the 1964 Helsinki Declaration and its later amendments or comparable ethical standards. Informed consent was obtained from all individual participants included in this study. Ethical approval was given by the Research Ethical Committee of Semmelweis University (172/2022).

Reports and MR images of all patients from our UAE database containing 801 patients who had UAE in the period between April 1, 2008, and September 30, 2021, were reviewed by a senior radiology resident (KT) and validated by a certified radiologist (IK) with more than 20 years’ experience in female pelvic MR to identify patients with pure adenomyosis. All patients who had preprocedural MRI examination where the images were still available were enrolled. Post-UAE MR imaging for the pure adenomyosis patients was performed at 7.6 ± 3.8 months.

MR imaging was performed with 1.5 T and 3.0 T systems (Philips Ingenia 1.5 T, Philips Achieva 1.5 and 3.0 T, and Siemens Magnetom Harmony 1.0 T). MRI protocol contained T1, T2, and T1 contrast-enhanced sequences.

**Fig. 1:**
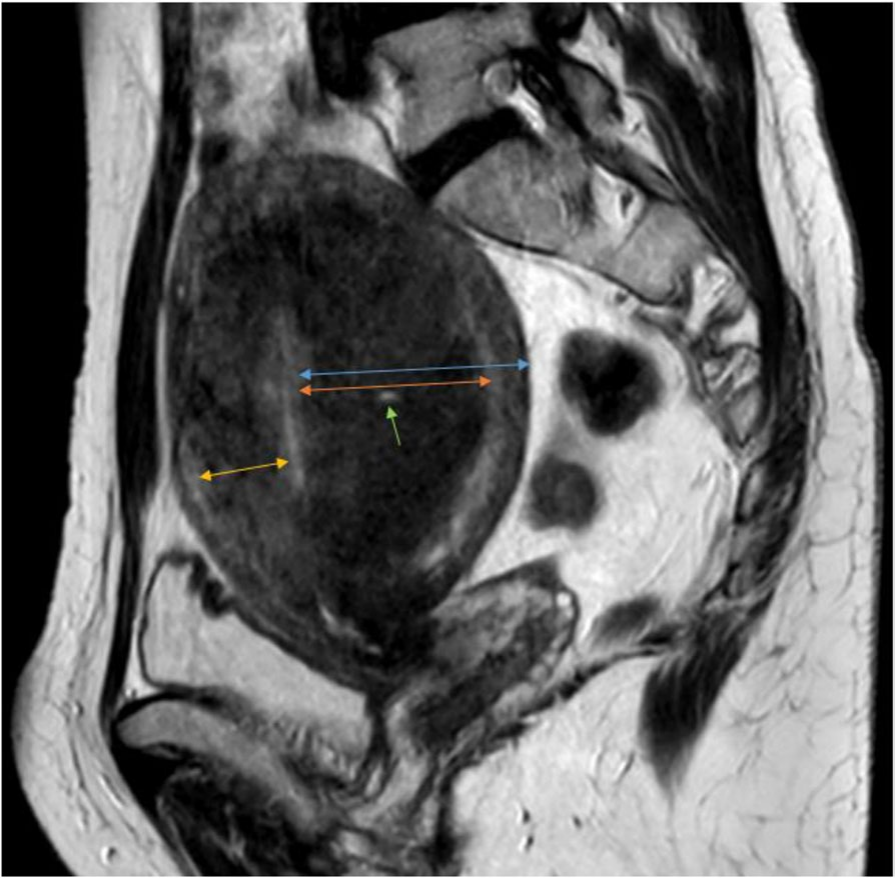
Sagittal T2W image of a 50-year-old patient. JZmax (orange arrow) is 42 mm. JZmin (yellow arrow) is 20 mm. JZdiff is 22 mm in this case (JZmax-JZmin). Myometrial thickness at the point of JZmax (blue arrow) is 55 mm. JZratio is 0.76 (JZmax/ myometrial thickness at the point of JZmax). The green arrow marks a T2 hyperintense focus within the thickened junctional zone. The radiological diagnosis of uterine adenomyosis is evident

Adenomyosis was diagnosed if T1 or T2 hyperintense submucosal microcysts were detected as the principal direct sign of the disease [4]. Adenomyosis was also diagnosed if the maximal junctional zone thickness (JZmax) was greater than 12 mm. In the case of a JZmax measurement between 8 and 12 mm, the diagnosis was given if the JZdiff was higher than 5 mm and the JZratio was higher than 0.4 [12, 13]. Junctional zone differential (JZdiff), and junctional zone ratio (JZratio) were also obtained in each case [6]. The minimal thickness was measured on the anterior and posterior uterine walls; for the calculation of the JZ differential, the smaller value was used regardless of which wall the maximal JZ thickness was measured (Fig. 1).

Differences between pre- and postprocedural values of uterine volume (Uvol), JZmax, JZdiff, and JZratio were obtained. Non-perfused volume on contrast-enhanced T1 sequences following embolisation were categorized as total, partial, or none. Preprocedural MRI morphological parameters (JZmax, JZdiff, JZratio, Uvol) were correlated with the pre-UAE QoL score. Correlation between UAE-induced change in MRI morphological parameters and change in QoL score was also analysed. Pre-UAE MRI morphological parameters and change in QoL score were correlated.

All embolisation procedures were performed using standard procedures by the same interventional radiologist (VB), who had more than 20 years of experience. A catheter was inserted using the unilateral right common femoral artery access, and super-selective angiography of both uterine arteries was obtained with a 4F catheter. Embolisation was achieved by injecting non-spherical polyvinyl alcohol (PVA) particles into each uterine artery (500–710 μm, COOK PVA-500, Bloomington, IN, USA; 500–700 and 355–500 μm Contour, Boston Scientific-Target Therapeutic, Fremont, CA, USA) until it reached a near-stasis flow state. The volume of injected PVA was noted. Volume of injected PVA, preprocedural MRI morphological parameters, and QoL score change were correlated. The puncture site was manually compressed after the procedure. Patients routinely received antibiotic prophylaxis (amoxicillin-clavulanic acid or clindamycin). Tramadol, meloxicam, metamizole-Na, nalbuphine, and drotaverine were additionally given for postoperative pain control.

At our centre clinical success was routinely assessed by interviews where patients were asked whether their symptoms had improved, had improved partially, or had not improved; and if they would recommend UAE to other patients with symptomatic adenomyosis. A numerical analogue quality-of-life (QoL) score (0: intolerable symptoms, 100: perfect QoL) was obtained before and after the embolisation in all patients to assess the clinical efficacy of the procedure [14, 15]. A difference between pre- and postUAE values of QoL was obtained. QoL change was calculated based on the preprocedural and postprocedural (1 year after UAE) QoL score. Long-term clinical follow-up for QoL score was also analysed. Complications were documented. Follow-up time ended by the last office meeting/telephone interview, or at the time of menopause or elective hysterectomy.

Data are expressed as median and range. Wilcoxon signed-rank test, uni- and multivariate regression models, Pearson product-moment correlation, and Kruskal–Wallis tests were used for statistical analysis (R Statistical Software, v4.1.2; R Core Team 2021). P values < 0.05 were considered statistically significant.

## Results

From our database of all UAE patients (801 patients between April 2008 and September 2021), 577 patients had available MR reports and images before UAE. Pre- and post-UAE MR images and reports were available in 420 cases. Pure adenomyosis without uterine fibroids was identified in 15 cases; 12 of 15 patients had pre- and post-procedural MRI. The mean age of patients at the time of UAE was 44.4 ± 5.4 years. The mean value of the total amount of injected PVA during UAE was 3.0 ± 1.5 ml. We found significant correlation between injected PVA volumes and Uvol before the procedure and with JZmax before the procedure (p = 0.03 and 0.02 respectively). We found no significant correlation between injected PVA volumes and QoL change.

No significant correlation could be observed between preprocedural MRI morphological factors and preprocedural QoL score, or QoL score change.

**Fig. 2:**
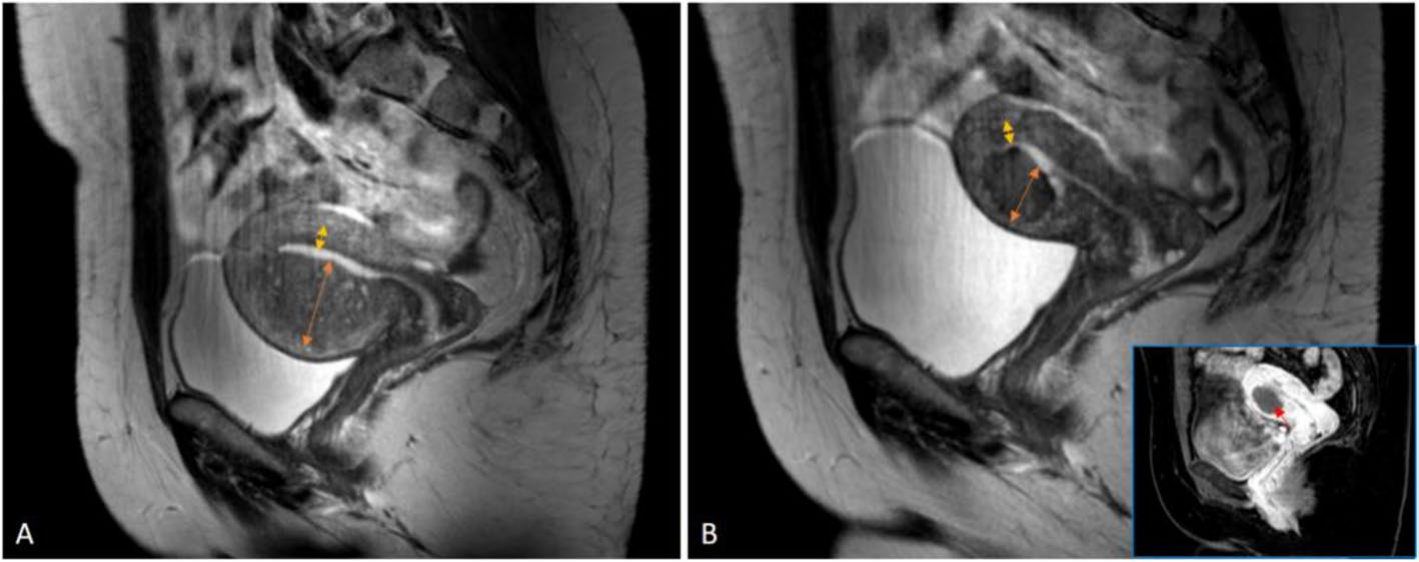
Preprocedural (**A**) and postprocedural (**B**) sagittal T2W images of a 49-year-old patient. The postprocedural examination was obtained 6 months after the UAE. Inserted sagittal contrast-enhanced T1-weighted image shows a necrotic area without contrast enhancement (red arrow on inserted image). JZmax (marked by an orange arrow on both images) was 35 mm before, and 21 mm after UAE. The minimal junctional zone thickness (JZmin) is marked by a yellow arrow on both images. JZdiff (JZmax-JZmin) was 33 mm before, and 16 mm after UAE. JZratio (not marked on images) was 0.88 and 0.84 before and after UAE, respectively. Uterus volume (not marked on the images) was 182 cm3 and 90 cm3 before and after UAE, respectively

Pre- and post-procedural MR parameters are presented in Table 1: Uvol, JZmax, and JZdiff decreased significantly after UAE (*p* = 0.003, 0.013, and 0.023, respectively); there was no significant change in JZratio (*p* = 0.5529). A representative case is shown in Fig. 2.

**Table 1:** MRI parameters before and after UAE. Wilcoxon signed-rank test were used for statistical analysis. A negative value in difference implies an increase

	Before		After		Difference in percentage		Difference (*p* values)
	Median	Range	Median	Range	Median	Range	
Uterus volume (cm3)	298	612	182	624	26%	48%	0.0025
JZmax (mm)	41	59	29	35	11%	62%	0.0133
JZdiff (mm)	33	60	21	39	12%	85%	0.0226
JZratio	0.88	0.29	0.92	0.26	-0.02%	0.4%	0.5529

A significant correlation was found between QoL change and JZmax change (p = 0.005, r = 0,776), and between QoL change and JZdiff change (p = 0.016, r = 0,704), while no significant correlation could be found between QoL change and uterine volume change and between QoL change and JZratio change.

Non-perfused volume was total in 1 case (1/12, 8.3%), partial in 5 cases (5/12, 41.7%), and none in 6 cases (6/12, 50%). QoL score significantly improved both in the “partial” (QoL score change 81 ± 19, p = 0.029)—and “none” (QoL score change 61 ± 27, p = 0.031) groups; there were no significant differences between the “partial” and “none” groups in the pre-UAE QoL score, in the post-UAE QoL score, or in the QoL score changes.

**Fig. 3:**
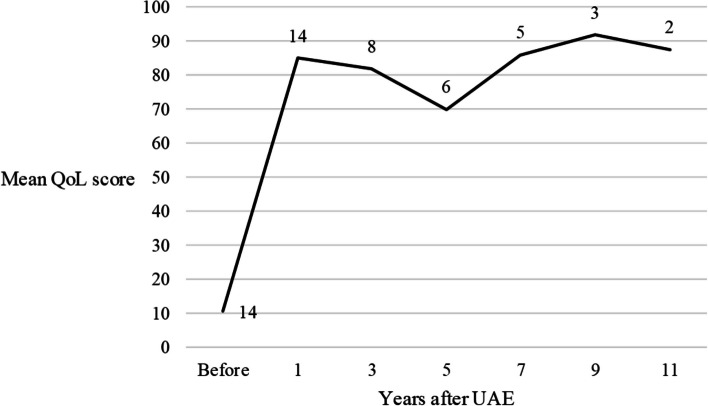
Long-term follow-up data on QoL score. Numbers on the curve indicate the number of patients

Clinical follow-up data are presented in Table 2. Clinical data, changes in QoL score, and any data on complications were available from 14 patients (14/15, 93.3%). All patients reported clinical success, and at least partial improvement in their symptoms (14/14, 100%; n = 12 “Yes”, n = 2 “partially), and all patients indicated that they would recommend UAE to other patients (14/14, 100%). The mean follow-up time was 64.6 ± 46.5 months (range: 9–147 months). QoL score increased significantly 1 year after UAE (p = 0.001) and the increase was permanent up to 11 years (Table 2; Fig. 3). Permanent amenorrhoea was observed in one 47-year-old patient within 1 year (1/14, 7.1%); this patient considered menopause at this age as an advantage and not as a complication. For one 44-year-old patient, elective hysterectomy was performed 5 years after UAE (1/14, 7.1%).

**Table 2:** Short- and long-term clinical data. Wilcoxon signed-rank test was used for statistical analysis in case of short-term data. Kruskal–Wallis rank sum test was used for statistical analysis in case of long-term data. P values represent no significant change between the QoL score 1 year after UAE and the QoL score 3/5/7/9/11 years after UAE

	Median	Range	*p* values	Number of patients
Short-term				
QoL score before UAE	5	30	0.0011	14
QoL score 1 year after UAE	95	70		14
Difference in QoL score before and after UAE	75	70		
Long-term				
QoL score 3 years after UAE	97.5	70	1	8
QoL score 5 years after UAE	77.5	70	0,2012	6
QoL score 7 years after UAE	95	40	0,4227	5
QoL score 9 years after UAE	95	20	1	3
QoL score 11 years after UAE	87.5	15	1	2

## Discussion

### Morphological data

Multiple papers showed a decrease in uterus volume of 8–54% within a year following UAE, assessed by MRI [9]. Our data on uterine volume decrease in cases of pure adenomyosis (median 26%, range 48%) falls within the range reported in the literature.

The decrease in JZmax following UAE was 12.0–33% in the studies published 1999–2010 [10]. In the other systematic review of studies between 2001–2016, 4 papers reported a decrease in JZ of 13.7%–38%; however, most papers (26/30, 86.7%) cited in this systematic review did not report on JZ [9]. JZ thickness was shown to be thicker in patients who underwent hysterectomy for persistent symptoms [14]; however, the insufficient response was not correlated with significantly thicker JZ in another paper [15]. Our finding of a 15% mean reduction in JZ thickness is in line with previous reports.

Besides the widely used junctional zone thickness, JZ differential and JZ to myometrial ratio have also been suggested as objective measures to improve the diagnostic accuracy of MRI in adenomyosis [6, 13, 16]. Kitamura et al. found no significant decrease in JZratio after embolization of 19 patients with pure or dominant adenomyosis [11] – our findings regarding JZratio change are in alignment.

This is the first study to our knowledge to report on the correlation between QoL change and junctional zone characteristics. A significant correlation between QoL change vs. JZmax change, and QoL change vs. JZdiff change underlines the importance of these measurements and their relation to the imaging appearance of adenomyosis. JZmax as the classic, long-debated MR morphological factor still in use in everyday practice, forms the base of the imaging diagnosis of adenomyosis and is a main pillar in some aspects of disease classification [13, 16, 17]. JZdiff, on the other hand, is considered as a second-line measurement in imaging diagnosis. Based on our findings, JZdiff could play a more significant role in the imaging diagnosis and the ever-evolving classification of adenomyosis as a marker of junctional zone inhomogeneity. Moreover, the change in JZdiff after UAE in adenomyosis may be a potential marker of clinical success.

The infarction rate of adenomyosis following UAE, with a reported range of 44.2%-82.5%, seems to be smaller than that of fibroids; however, most studies (26/33, 78.8%) did not report the infarction rate [9]. Bae et al. concluded that an infarction cut-off rate of < 34.4% had a 7 times higher risk of symptom recurrence [18]. One other study, however, showed that correlation between improvement of symptoms and imaging at 3-month follow-up was statistically not significant [19]. Our results resonate with this latter paper: there was a significant improvement in QoL score in both the”none” and “partial” groups; in addition, symptom improvement (increase in QoL sore) was not significantly different between the”none” and”partial” groups, suggesting that total infarction of the adenomyotic region is not a prerequisite for clinical success.

The exact mechanisms of heavy menstrual bleeding and dysmenorrhoea in adenomyosis, and of how UAE improves symptoms in adenomyosis, are not fully understood. Microvessel density, endometrial surface, and overall uterine size may be contributing factors [11, 20]. Following embolisation, as opposed to normal myometrium, adenomyotic tissue presumably is not capable of opening up vessels; thus, it may be less tolerant to ischaemia [20, 21].

### Clinical data (patient satisfaction, short-term and long-term clinical success, complications)

The overall patient satisfaction rate in the systematic review by Popovic et al. was 75.7% [10]. The more recent systematic review reported patient satisfaction rates for < 12 months follow-up for pure adenomyosis and combined adenomyosis as 89.6% and 94.3%, respectively; long-term data (> 12 months) for pure adenomyosis and combined adenomyosis was 74.0% and 85.4%, respectively. A recent study showed 90% clinical success [20]. The longest follow-up (95 ± 9 months) was reported by de Bruijn et al. [22]. In our study the second-longest follow-up data (mean follow-up time was 65 ± 47 months [median 55, range: 9–147 months]) on patients with pure adenomyosis showed 100% patient satisfaction (symptom improvement”yes” in 85.7%,”partial” in 14.3%), and all patients in our study would recommend UAE to another patient with adenomyosis.

Clinical follow-up in the literature is mostly provided as symptom improvement; UFS-QoL scores (n = 5) or HRQOL (n = 1) were analysed only in 20.0% (6/30) of the studies, showing significant improvements in UFS-QOL [9]. Uterine fibroid questionnaires were used since adenomyosis-specific QoL questionnaires were not available. We used numerical analogue QoL score to assess clinical efficacy. This score was used in previous publications [23, 24] to assess clinical effectiveness of UAE in symptomatic uterine fibroids; however, this score is not specifically for fibroid symptoms. Here it provides a score for the clinical improvement of pre-UAE symptoms.

Popovic showed that not all studies reported on complications; among the reported cases, 13.2% (1.5 to 29.3%) had a hysterectomy, with the majority conducted approximately 12 months (range 2–27 months) after UAE (10). In the review by de Bruijn from 2017, only 20/30 (66.6%) studies reported on complications: hysterectomy in the short-term (< 12 months) follow-up group for pure adenomyosis and combined adenomyosis was 2.6% and 1.4%, respectively [9]. The same numbers for long-term (> 12 months) follow-up were 7.2 and 7.0%, respectively. The hysterectomy rate was 18% in the 7-year follow-up group (22). In our cohort, the hysterectomy rate was 7.1% with the second longest follow-up period of 65 ± 47 months.

In the systematic reviews, permanent amenorrhoea occurred in 20.9% of patients, all whom were > 45 years old (10); it was 6.3% in those, > 40 years old (3). Our data showed similar results (7.1%) at the mean age of 47 years.

Other published complications included spontaneous fibroid expulsion in 10 patients, and four suspected cases of endometritis; all responded well to broad-spectrum antibiotics. Deep venous thrombosis in the calf was reported in one case (9). In the study of the longest (7-year) follow-up, 36% of the patients experienced the absence of menstrual periods for at least 12 months [22]. There was no deaths or serious adverse events (including emergency hysterectomy) [9, 10].

### Strengths and limitations

This is the first study to our knowledge to report on the correlation between QoL change and junctional zone characteristics in adenomyosis after UAE with the second-longest clinical follow-up.

The main limitations are the low number of patients in this single-centre, retrospective study and the long period over which the procedures were performed.

## Conclusion

Our results indicate that UAE is safe and clinically effective for up to 11 years in the treatment of pure adenomyosis. However, a randomised controlled trial confirmation would be essential to reinforce our findings; accordingly data from the QUESTA trial are keenly awaited [25]. The level of non-perfused areas seems not to be a prerequisite to clinical improvement. JZdiff as a marker of junctional zone inhomogeneity could play a more significant role in the MR imaging diagnosis of adenomyosis. Changes in JZmax and JZdiff after UAE in adenomyosis are potential markers of clinical success. Further research is needed with a larger number of patients to establish the potential role of the marker and test its relationship with other junctional zone characteristics.

## Data Availability

The datasets used and/or analysed during the current study are available from the corresponding author on reasonable request.

## Abbreviations

JZ: Junctional zone
JZdiff: Junctional zone differential
JZmax: Maximal junctional zone thickness
JZratio: Junctional zone ratio
HRQOL: Health-related quality of life
MRI: Magnetic Resonance Imaging
PVA: Polyvinyl alcohol
QoL: Quality of life
UAE: Uterine artery embolisation
UFS-QOL: Uterine Fibroid Symptom and Health-related Quality of Life
Uvol: Uterine volume

## References

[1] Kim MD, Kim YM, Kim HC (2011). Uterine artery embolization for symptomatic adenomyosis: a new technical development of the 1–2-3 protocol and predictive factors of MR imaging affecting outcomes. J Vasc Interv Radiol.

[2] Lohle PNM, Higué D, Herbreteau D (2019). Uterine artery embolisation in women with symptomatic adenomyosis. Presse Med.

[3] Gordts S, Grimbizis G, Campo R (2018). Symptoms and classification of uterine adenomyosis, including the place of hysteroscopy in diagnosis. Fertil Steril.

[4] Novellas S, Chassang M, Delotte J (2011). MRI characteristics of the uterine junctional zone: from normal to the diagnosis of adenomyosis. AJR Am J Roentgenol.

[5] Celli V, Dolciami M, Ninkova R (2022). MRI and adenomyosis: what can radiologists evaluate?. Int J Environ Res Public Health..

[6] Rees CO, Nederend J, Mischi M, van Vliet HAAM, Schoot BC (2021). Objective measures of adenomyosis on MRI and their diagnostic accuracy-a systematic review & meta-analysis. Acta Obstet Gynecol Scand.

[7] Ma J, Brown B, Liang E (2021). Long-term durability of uterine artery embolisation for treatment of symptomatic adenomyosis. Aust N Z J Obstet Gynaecol.

[8] Mailli L, Patel S, Das R (2023). Uterine artery embolisation: fertility, adenomyosis and size - what is the evidence?. CVIR Endovasc..

[9] de Bruijn AM, Smink M, Lohle PNM (2017). Uterine artery embolization for the treatment of adenomyosis: a systematic review and meta-analysis. J Vasc Interv Radiol.

[10] Popovic M, Puchner S, Berzaczy D, Lammer J, Bucek RA (2011). Uterine artery embolization for the treatment of adenomyosis: a review. J Vasc Interv Radiol.

[11] Kitamura Y, Allison SJ, Jha RC, Spies JB, Flick PA, Ascher SM (2006). MRI of adenomyosis: changes with uterine artery embolization. AJR Am J Roentgenol.

[12] Agostinho L, Cruz R, Osório F, Alves J, Setúbal A, Guerra A (2017). MRI for adenomyosis: a pictorial review. Insights Imaging.

[13] Chapron C, Vannuccini S, Santulli P (2020). Diagnosing adenomyosis: an integrated clinical and imaging approach. Hum Reprod Update.

[14] Smeets AJ, Nijenhuis RJ, Boekkooi PF, Vervest HA, van Rooij WJ, Lohle PN (2012). Long-term follow-up of uterine artery embolization for symptomatic adenomyosis. Cardiovasc Intervent Radiol.

[15] Nijenhuis RJ, Smeets AJ, Morpurgo M (2015). Uterine artery embolisation for symptomatic adenomyosis with polyzene F-coated hydrogel microspheres: three-year clinical follow-up using UFS-QoL questionnaire. Cardiovasc Intervent Radiol.

[16] Bazot M, Daraï E (2018). Role of transvaginal sonography and magnetic resonance imaging in the diagnosis of uterine adenomyosis. Fertil Steril.

[17] Kobayashi H, Matsubara S (2020). A classification proposal for adenomyosis based on magnetic resonance imaging. Gynecol Obstet Invest.

[18] Bae SH, Kim MD, Kim GM (2015). Uterine artery embolization for adenomyosis: percentage of necrosis predicts midterm clinical recurrence. J Vasc Interv Radiol.

[19] Jha RC, Takahama J, Imaoka I (2003). Adenomyosis: MRI of the uterus treated with uterine artery embolization. AJR Am J Roentgenol.

[20] Liang E, Brown B, Rachinsky M (2018). A clinical audit on the efficacy and safety of uterine artery embolisation for symptomatic adenomyosis: Results in 117 women. Aust N Z J Obstet Gynaecol.

[21] Antero MF, Ayhan A, Segars J, Shih IM (2020). Pathology and Pathogenesis of Adenomyosis. Semin Reprod Med.

[22] de Bruijn AM, Smink M, Hehenkamp WJK (2017). Uterine artery embolization for symptomatic adenomyosis: 7-year clinical follow-up using UFS-Qol questionnaire. Cardiovasc Intervent Radiol.

[23] Kalina I, Tóth A, Valcseva É, et al. Prognostic value of pre-embolisation MRI features of uterine fibroids in uterine artery embolisation. Clin Radiol. 2018;73(12):1060.e1–7. 10.1016/j.crad.2018.08.009. 10.1016/j.crad.2018.08.009 30309632

[24] Bérczi V, Valcseva É, Kozics D, et al. Safety and Effectiveness of UFE in Fibroids Larger than 10 cm. Cardiovasc Intervent Radiol. 2015;38(5):1152–6. 10.1007/s00270-014-1045-4. 10.1007/s00270-014-1045-4 25571883

[25] de Bruijn AM, Lohle PN, Huirne JA (2018). Uterine artery embolization versus hysterectomy in the treatment of symptomatic adenomyosis: protocol for the randomized QUESTA trial. JMIR Res Protoc..

